# The ethics of future trials: qualitative analysis of physicians’ decision making

**DOI:** 10.1186/s13063-015-1137-8

**Published:** 2016-01-06

**Authors:** Fiona Webster, Charles Weijer, Laura Todd, Jeremy M. Grimshaw, Andrea P. Marshall, Deborah Cook, Graeme MacLennan, Brian H. Cuthbertson, Jill J. Francis, for the SuDDICU international group

**Affiliations:** Department of Family and Community Medicine, University of Toronto, 500 University Avenue, 5th Floor, Toronto, Ontario M5G 1V7 Canada; Rotman Institute of Philosophy, Western University, 1151 Richmond Street, London, Ontario N6A 5B8 Canada; Clinical Epidemiology Program, Ottawa Hospital Research Institute, Ottawa, Canada; Department of Medicine, University of Ottawa, Ottawa Hospital - General Campus, Centre for Practice-Changing Research, 501 Smyth Road, Room 1286, Ottawa, Ontario K1H 8L6 Canada; Menzies Health Institute Queensland, Griffith University and Gold Coast University Hospital, D.5 090 1 Hospital Blvd, Southport, QLD 4215 Australia; Departments of Medicine, Clinical Epidemiology & Biostatistics, McMaster University, Faculty of Health Sciences, 1280 Main Street West, Rm. 2C11, Hamilton, Ontario L8S 4K1 Canada; Health Services Research Unit, Health Sciences Building, Foresterhill, University of Aberdeen, Aberdeen, AB25 2ZD UK; Department of Critical Care Medicine, Sunnybrook Health Sciences Centre, Toronto, Canada; Department of Anesthesia, University of Toronto, Toronto, Canada; School of Health Sciences, City University London, Northampton Square, London, EC1V OHB UK

**Keywords:** Ethics, Clinical equipoise, Harm-benefit analysis, Critical care, Evidence-based medicine, Qualitative research, Randomized controlled trials

## Abstract

**Background:**

The decision to conduct a randomized controlled trial (RCT) in a field raises ethical as well as scientific issues. From the clinical equipoise literature, future trials are justifiable if there is ”honest, professional disagreement in the community of expert practitioners as to the preferred treatment”. Empirical data are sparse about how clinicians apply the principles of equipoise to the justification of future RCTs. For example, selective decontamination of the digestive tract (SDD) is not widely used in critical care practice despite the strength of the evidence base and therefore provides a unique opportunity to learn how clinicians think about the ethics of further RCTs in critical care.

**Methods:**

In an international interview study of views of healthcare professionals about SDD, we undertook a secondary analysis of qualitative data collected using a Theoretical Domains Framework of clinical behaviour. We adopted a general descriptive approach to explore how physicians determined whether another RCT of SDD is ethical. Following a constant comparison approach, three investigators reviewed 54 purposively chosen transcripts from three international regions. We interpreted the data using thematic analysis.

**Results:**

We grouped participants’ responses into four inter-related themes: 1) cultural norms about evidence and practice within healthcare; 2) personal views about what evidence is current or applicable; 3) the interpersonal and relational nature of professional decision making locally; and 4) an a priori commitment to future trials. The analysis also identified several unresolved tensions regarding when a future RCT should be pursued. These tensions focused on a clash between potential benefits to current individual patients and potential future harms to patients more broadly.

**Conclusions:**

Our study suggests that ethical decision making about future RCTs in the field of SDD does not rely strongly on appeals to evidence, even when the quality of the evidence is reasonably high. Rather, “extra-evidential” reasons, including social, professional, and relational factors, seem to influence opinions regarding the ethics of future trials. Further work is required to see if these conclusions are applicable to other clinical topics and settings.

**Electronic supplementary material:**

The online version of this article (doi:10.1186/s13063-015-1137-8) contains supplementary material, which is available to authorized users.

## Background

Randomized controlled trials (RCTs) help to provide reliable information on the safety and efficacy of healthcare interventions. To have scientific and clinical utility, an RCT of a novel intervention may be considered to have a “window of opportunity” with respect to the accumulating knowledge base. If little evidence exists at the proof-of-principle level, a large definitive RCT may be premature, and it may be unjustifiable to expose patients to a potentially ineffective (or even harmful) treatment in a subsequent trial. On the other hand, if the safety, efficacy, and effectiveness of an intervention have been rigorously established, a further RCT seems unlikely to contribute meaningfully to knowledge. Unnecessary repetition of RCTs is a more common problem than one might think. Fergusson and colleagues describe a series of 64 RCTs of aprotinin versus placebo to reduce preoperative transfusion that were conducted over a 15-year period [1]. This meta-analysis identified that the estimate of effectiveness stabilized at the twelfth study and demonstrated reduced bleeding with aproptinin. After this study, nearly 3,000 further patients were randomized to control groups in subsequent RCTs and were thus denied a treatment that at that time was of proven effectiveness.

The timing of initiation of an RCT raises ethical as well as scientific issues. Physician-researchers are widely regarded as having a duty of care to patients in RCTs, meaning that there must be good grounds to believe that study interventions are consistent with standards of medical care [2]. This ethical requirement, known as “clinical equipoise”, demands that, at the start of an RCT, there be a state of honest, professional disagreement in the community of expert practitioners as to the preferred treatment [3]. Yet, it is unclear which factors contribute legitimately to the professional disagreement central to clinical equipoise. Some argue that physicians themselves must be uncertain as to the preferred treatment, while others argue that the evidence supporting or refuting a treatment must itself be incomplete or uncertain [4]. Surprisingly little is actually known about how clinicians determine whether an RCT is ethical, and the factors to which they appeal in making this determination.

Our study context is the controversy surrounding the role of selective decontamination of the digestive tract (SDD) in preventing serious infections and mortality in the critically ill [5]. Ventilator-associated pneumonia (VAP) and other serious healthcare-associated infections are important causes of morbidity and mortality in intensive care units (ICUs). SDD involves the application of topical antibiotics to the oropharynx and stomach combined with a short course of intravenous antibiotics. Evidence supporting the effectiveness of SDD includes 37 RCTs (involving more than 7000 patients) that have been summarized in 12 meta-analyses and demonstrates that SDD reduces VAP (odds ratio (OR) = 0.28; 95 % confidence interval (CI) = 0.20–0.38) and mortality (OR = 0.73; CI = 0.64–0.84) [6–8]. (A comprehensive overview of the evidence supporting SDD may be found in a recently publication by Price and colleagues [8].) Despite such strong evidence, however, recommendations in professional guidelines are inconsistent, and SDD is not widely used in practice. A UK survey revealed that SDD was used in only 10–15 of 240 ICUs [9]. Clinicians may be reluctant to adopt SDD because of concerns about antibiotic resistant organisms, the applicability of RCTs conducted in other countries, or skepticism about its effectiveness [10].

The controversy over SDD thus provides a unique opportunity to explore how clinicians think about the ethics of future RCTs. The disparity between the strength of the evidence in favour of SDD, and the reluctance to use it in clinical practice, is stark. It is unclear that further trials would provide further clarity about clinical effectiveness. We asked the following research questions: How do clinicians determine whether another RCT of SDD is ethical? In making this determination, do they appeal to the evidence base, clinical practice, opinion, or other factors?

## Methods

Cuthbertson and colleagues studied the perceived risks, benefits, and barriers to the use of SDD in ICUs in Australia, Canada, and the UK [11]. We undertook a secondary analysis of SuDDICU (Selective Decontamination of the Digestive Tract in the ICU) clinician interview data using a general description approach [12–14]. Ethics approval for this secondary analysis was obtained through the University of Toronto Research Ethics Board; written consent had been collected prior to each interview in the primary study and was not required again for this analysis. Clinicians, including 141 ICU physicians, ICU pharmacists, ICU clinical leads, clinical microbiologists, and infectious disease specialists, were asked how they perceived the effectiveness of SDD and whether further research, including additional RCTs, was required [15]. The original data were collected in a semi-structured interview utilizing the Theoretical Domains Framework, facilitated by a topic guide developed by the multi-disciplinary research team using methods described elsewhere [11, 16]. Our study met the criteria for re-usability of qualitative data set out by Hinds in accordance with the key dimensions of: 1) accessibility (we conducted the original research); 2) quality (the original research design was publishable, the data set was complete, and a full summary was made of all analysis meetings held over a 12-month period); 3) suitability (the selected participants matched the emerging themes we identified and additional interviews were not required to achieve theoretical saturation) [17].

Using Braun and Clarke’s thematic approach, three researchers (FW and CW and LT) independently reviewed an initial series of nine purposively chosen transcripts from Canadian clinicians and then met to establish a coding framework [18]. LT then coded additional transcripts using this framework. This is a secondary analysis so, for the analysis reported here, recruitment was already completed at the study sites. The team concluded that saturation had been reached at 18 interviews within the Canadian data set [19]. A decision was made by the team to extend our analysis to include 18 transcripts each from the UK and Australia (with six participants from each of three professional groups: critical care physicians, pharmacists, and infectious disease specialists) where interviews were conducted over the same time period using the same interview guide. Our approach to sampling was based on maximum variation sampling, which involves selecting typical, atypical, and “information-rich cases” [20]. Researchers involved in data collection at the Canadian, Australian, and UK sites were asked to identify transcripts that seemed representative as well as any outliner accounts or transcripts that provided particularly information rich cases of inter-professional collaboration and decision making in relation to scientific evidence. We extrapolated the number of 18 required to reach saturation from the Canadian data set to the other data sets. Given nurses’ consistent statements that they were not involved in decisions about trial participation, they were not represented in this data set.

After data coding, we then focused our interpretation to identify similarities and differences across the interviews. This step involved combining codes into themes and searching for patterns. Researchers’ comments were recorded as marginal notes during six meetings in the coding/theme development phase to assist focus around emerging concepts. The analysis was between interpretations (CW, FW, and LT) that were captured in notes taken after every meeting. More than one investigator performed each step during this analysis. We discussed our biases during data analysis and recorded these conversations through the use of investigator memos to help ensure that our analysis was reflexive [21].

## Results

We analyzed 54 transcripts in this study, from interviews with 18 critical care physicians, 18 pharmacists, and 18 infectious disease specialists from Australia, Canada, and the UK. In our study, clinicians frequently described the factors external to the evidence that contributed to their decision making regarding the ethics of future RCTs. We grouped their responses into four inter-related themes: 1) cultural norms about evidence and practice within healthcare practice, including a belief about the infallibility of guidelines; 2) personal views about what evidence is current or applicable; 3) interpersonal and relational aspects of professional decision making locally; and 4) an a priori commitment to future trials testing SDD. Each of these factors was identified by participants as influencing their uptake of evidence and decision making regarding the ethics of future SDD trials. (See Additional file 1: Table S1 for additional quotes.)

### Theme 1: cultural norms within healthcare practice

Participants’ conceptualization and understanding of the evidence, and how they utilized it in their own decision making, revealed several strongly articulated cultural norms within critical care about evidence and practice. While clinicians adhere publicly to the concept of evidence-based medicine (EBM), those in our study explained that evidence alone is insufficient to determine practice. Views about the use of evidence did not vary by geographic location or profession. For example:

“… *physicians are a strange bunch, because they’ll all tell you that they want to see evidence before adopting a practice, and yet often they do things that… go against evidence or… are based on sort of one-off experiences they’ve had… I think physicians are a little bit difficult in that way.”* (Infectious Disease Physician, Canada, 025)

The reasons given by participants for not following evidence varied but were seldom based on methodological criticism of supporting evidence or appeal to contradictory evidence. For some, resistance to evidence was related to maintaining one’s professional reputation. One critical care physician described how adopting new evidence might call into question the validity of previous practices, suggesting that:

*“… people [might] now not be willing to change their mind regardless of the evidence because to do so at this point would almost undermine all the years that they actually said it didn’t work.”* (Critical Care Physician, UK, 210)

Many interviewees also described regularly following practices which were not evidence-based. One participant cited collegial agreement and potential lack of harm, to justify engaging in practices for which there is “*quite poor evidence*”:

*“the head of bed elevation…even though [it has] quite poor evidence … and especially even when compared to SDD, people will say, ‘Well, what’s the big deal… in just elevating the head of the bed? It’s an easy thing to do’… the science behind it’s bad, but people just agree….”* (Critical Care Physician, Canada, 043)

Another cultural norm that emerged from our interviews was deference to guidelines. Many participants were unwilling to trust their own critical appraisal of the evidence if this meant going against norms or guidelines. As one participant argued:

*“I think because the national and international bodies haven’t come out… jumping up and down and saying it’s a good thing to do that everyone’s a bit leery to strike out on their own”.* (Critical Care Physician, Canada, 315)

In relation to the evidence for SDD specifically, some physicians felt that there was strong evidence to adopt SDD yet similarly strong resolve to not adopt it:

*“it’s been very interesting that it’s not adopted, yet evidence would suggest it should be and, and it’s almost actively not adopted if you know what I mean. It’s the … best example we have against evidence-based medicine. Despite a level of evidence we’re happy to apply to other things … it still isn’t incorporated into practice…”* (Critical Care Physician, Australia, 210)

### Theme 2: personal views about what ideas are current or applicable

For some participants, their personal view that the effectiveness of SDD is “old news” was a salient factor in their decision making. Although evidence is meant to be cumulative, in practice it turns out that the interpretation of evidence might more often be cyclical. In the current climate of rapidly produced new evidence, “old news” evidence might not be implemented despite its solid grounding in science. As one participant noted:

*“I think [there have] been various iterations of SDD over the years and it’s come and gone in terms of fashion. I remember in 1993 … it was quite fashionable to do it at that stage and then it went out of vogue and more recently it’s come back into vogue again and … has been a bit cyclical in its history.”* (Critical Care Physician, Australia, 044)

In addition, several participants considered that the results of trials that had been conducted in other countries were not relevant or applicable in their own. This held true even when patient populations and healthcare systems were relatively similar across countries. For example, one Canadian pharmacist indicated that he considered studies conducted in Europe to have “*different microbial flora, [and a] different patient population* (Pharmacist, Canada, 013), while a critical care physician in Australia believed that, before making a decision to implement as a *“standard of practice”*, they would need to see:

“*evidence replicated in a setting outside of Northern Europe, preferably in Australia”* (Critical Care Physician, Australia, 301)

### Theme 3: interpersonal and relational aspects of professional decision making locally

Participants in Australia and Canada expressed their perception that members of other professional groups had differing beliefs or priorities with respect to SDD. For example, some critical care physicians described their infectious disease colleagues as being singular in their “*concern about the possibility of resistant organisms*” and further indicated that in their hospital, “*antibiotic usage is controlled by the microbiologists …”* (Critical Care Physician, Australia, 304).

On the other hand, some of the infectious disease physicians explained their concerns about SDD and antibiotic resistance, concerns which they felt were overlooked by the intensivists::

“*The intensivists might be embracing this with open arms, but unfortunately the intensivists aren’t really concerned with anything outside the doors of the ICU… a disaster created in the ICU… becomes a disaster on the ward and the rest of the hospital, so the intensivists have to remember that what they do in their little area could have grave implications system-wide.”* (Infectious Disease Physician, Canada, 044)

Within their professional groups many participants insisted that decisions were made on a team basis:

*“And I think also we try where we can to do most things in a sort of … collegiate fashion with consensus rather than having one person implementing things.”* (Critical Care Physician, UK, 304)

Nonetheless, many participants also specified that one particular individual (often the head of a unit or department) had decision-making authority within their unit.

*“Because I’m external to the ICU the decisions on protocols are basically made by the director of the ICU … so if the director of our ICU felt very strongly in favour of something and we didn’t like it, you would do it anyway.* (Infectious Disease Physician, Australia, 405)

Still other participants seemed steadfast in their conviction that they would never adopt SDD.

*“[In order to adopt SDD in my unit] someone would have to assassinate me.”*

(Infectious Disease Physician, Canada, P10)

### Theme 4: an a priori commitment to future trials

Several participants expressed their belief that future trials were unnecessary or infeasible:

*“I don’t know how many times you have to beat people over the head with a stick for them to get it. I guess if there was to be more research, well, what would it be?… at some point I think people either have to buy into it or you have to move on to a different topic”.* (Critical Care Physician, Canada, 022)

Many participants clearly supported this opinion that a future trial was unnecessary, suggesting that such a trial would be:

*“just wasting everybody’s time because I think the people… have got their mind set up against it”* (Infectious Disease Physician, UK, 1704).

*“We do a lot of research but I guess I can’t really see how [testing implementation strategies] is research. It’s more implementation of a treatment which probably has evidence.”* (Critical Care Physician, Australia, 304)

Paradoxically, many of these same participants expressed a willingness to participate in future effectiveness trials:

*“I am not convinced there is overwhelming evidence that we should all be doing it because if there was overwhelming evidence that we should all be doing it, then we would all be doing it. Therefore I think there is still a potential for more research.”* (Pharmacist, UK, 3603)

*“Oh absolutely I think there’s, I think there’s equipoise on this issue and so [a trial] is absolutely justifiable.”* (Infectious Disease Physician, Australia, 401)

*“I think it [a trial] is ethical because there is a huge, I think there is equipoise within the medical profession.”* (Critical Care Physician, UK, 1701)

## Discussion

Our study found that the descriptions which clinicians provided of their ethical decision making and practice were significantly influenced by the “extra-evidential” factors described above (that is, cultural norms about evidence and practice within healthcare practice, including a belief about the infallibility of guidelines and personal biases about what evidence is current or applicable). Interestingly, these norms transcended professional and geographical boundaries. Physicians in our study did appeal directly to the concept of clinical equipoise but based their assessment of whether clinical equipoise obtains on socio-organizational types of factors, including practice norms, personal opinion, and clinical experience. Equipoise is a term frequently used in discussions of clinical trials. Yet, despite the discursive centrality of this concept in the ethical justification for clinical trials, there is a surprising lack of empirical research exploring physicians’ views and practices. The current study addresses this gap and provides evidence that such “extra-evidential” reasons influence ethical decision making to a much greater extent than previously recognized.

In our analysis we paid particular attention to inconsistencies within and between participants’ accounts of their use of evidence in clinical and ethical decision making. We found many statements that contradicted the notion that clinicians undertook an objective appraisal of scientific evidence when making decisions regarding the ethics of future RCTs on this topic. In general, participants described a widespread tendency to ignore scientific evidence and to accept published guidelines uncritically as a substitute for individual appraisal of evidence. This is particularly interesting, and may seem surprising in light of the longstanding problems related to variability in physician adherence to published guidelines and the difficulty in increasing guideline adherence [22].

The social context in which clinicians practice greatly influences their perceptions. In an important qualitative study by Donovan et al. [23], researchers found that “Many doctors acknowledged that they had ’hunches‘ or ’gut instincts‘ that particular treatments were superior in general or for specific patients or groups, and many experienced discomfort because of their clinical instincts and the ’blurring‘ of equipoise around rigid RCT eligibility criteria”. As noted above, evidence may also be perceived as cyclical rather than cumulative. Some practitioners referred to evidence supporting SDD as “old news” while others stated that implementing the evidence about SDD would first require personal “buy-in”. The role played by the clinical team in assessing evidence is complex and, at times, contradictory. Clinicians’ membership in specific subspecialties seemed to influence their interpretation of the evidence in relation to the relative importance of perceived benefits and harms. Infectious disease specialists, not unsurprisingly, focused on concerns about population antibiotic resistance rates, while critical care physicians expressed more concern for individual patient welfare. Moreover, physicians from one specialty spoke about their colleagues from another professional group in terms that seemed at times stereotypical, such as the notion that critical care physicians “aren't really concerned with anything outside the doors of the ICU”. Professional polarization thus had a significant impact on the way clinicians think about issues such as whether they should focus on the individual patient under their current care (the ethical principle of beneficence) or the well-being of future patients (the principle of justice). These questions of values cannot be resolved by appealing to scientific evidence alone.

A number of critics argue that the current thinking on EBM ignores the relational aspects of knowledge and dismisses the role of values in healthcare [22]. For such critics, “the clinician can often be considered… an institutional subject who is presumed both to know the truth of disease and to have the moral and intellectual authority to prescribe treatment” [24]. However, proponents of EBM have pointed out that values play a role in every important patient care decision [25]. Our qualitative study of clinicians’ reasoning about current SDD practice and future SDD trials highlights how decisions are sometimes consistent with individual values, or group values or societal values. The subjective and relational aspect of knowledge is particularly significant in accounts of how the ethics of future research are determined. Many individual clinicians demonstrate a type of “group think”, expressing a strong adherence to decisions made by the local team [26]. This supports our contention that there are interpersonal and relational influences involved in clinical reasoning that transcend the role played by individual reasoning. Contradictions were also apparent between how clinicians thought decisions ought to be made (by the team), and how they actually were made in practice (by a key individual who held a leadership position). We found these inconsistencies to persist across various professional disciplines and across the three international regions involved in our study. The majority of participants expressed their faith in EBM while acknowledging the prevalence of non-scientific factors in clinical decision making. As noted elsewhere, EBM is as much a discourse as an actual practice and, as our findings suggest, its widespread adoption tends to obscure practices that are not necessarily exclusively scientifically informed.

### Limitations

One of the limitations of secondary analysis of qualitative data is a lack of involvement in generating the original data set. Our original use of the Theoretical Domains Framework in the primary study, however, resulted in the generation of rich, in-depth data, which mitigated this challenge. Given the large number of interviews originally conducted, we were able to purposively select transcripts and believe that saturation was reached. We identified “key stakeholder groups” to participate in this study but did not include all possible stakeholders in our analysis. In addition, our choice of SDD as an example presents a case in which strong randomized trial evidence has not been adopted in practice. Therefore, it presents a special case, and our findings may not be generalizable to other interventions. Further limitations may include the study being performed in English-speaking countries only and in countries where SDD implementation is low.

## Conclusion

We sought to ascertain how clinicians decide whether future RCTs in a field are ethical. This study reveals that, regarding SDD, ethical decision making did not rely strongly on appeals to evidence, even when the quality of the evidence is quite high. Clinicians from different specialities and different geographical regions consistently described having been influenced by extra-evidential factors in their day-to-day practice and in their willingness to participate in future trials. These extra-evidential factors include social, professional, and relational contexts. When clinicians appeal to factors other than the evidence base, it can have a significant impact on their willingness to design, conduct, and enrol patients into future RCTs or may limit their willingness to implement “apparently proven” therapies into their practices. Further work is required to see if these conclusions are applicable to other clinical topics and settings.

## Additional file

Additional file 1: Table S1. Additional quotes. (PDF 178 kb)

## Abbreviations

CI: confidence interval
EBM: evidence-based medicine
ICU: intensive care unit
OR: odds ratio
RCT: randomized controlled trial
SDD: selective decontamination of the digestive tract
SuDDICU: selective decontamination of the digestive tract in the intensive care unit
VAP: ventilator-associated pneumonia
