# Supplementary material for: The ethics of future trials: qualitative analysis of physicians’ decision making
Source: Trials. 2016 Jan 6;17:12. doi: 10.1186/s13063-015-1137-8 (PMC4704430; doi:10.1186/s13063-015-1137-8)
Supplement: Additional file 1: Table S1. — Additional quotes. (PDF 178 kb) [file 13063_2015_1137_MOESM1_ESM.pdf]

Supplementary Table 1. Additional quotes

Theme 1: Cultural norms about evidence and practice within healthcare practice, including a belief about the infallibility of guidelines
“Yeah most people are scared of it. I mean you would be more familiar than I about the jokes that go on about this you know the triumph of opinion over evidence that sort of thing…”(Infectious Disease Physician, Australia, 401)<br><br>“The problem with clinicians and evidence is you can use the evidence to back your argument and if doesn’t suit your argument you ridicule the evidence.”(Critical Care Physician, UK, 1702)<br><br>“I don’t know maybe that is part of it, which makes people now not willing to change their mind regardless of the evidence because to do so at this point, would almost undermine all the years that they actually said it didn’t work.” (Critical Care Physician, UK, 210)<br><br>“Well yeah it’s sort of in practice and related to you know the positioning of the patient and, and things along those lines that’s all, all of which have their own controversies but are relatively easy to, easy and cheap interventions to get right.” (Infectious Disease Physician, Australia, 403)<br><br>“Well, I think some things get seen to be introduced into clinical practice with what appears to be a lot less evidence in their favour than SDD. And other things like SDD is a classic example, there seems to be a lot of evidence but for whatever reasons, psychological or whatever reason, people are unwilling to accept it and I think that is really fascinating” (Infectious Disease Physician, UK, 1704)<br><br>“You know, yes more research is needed for everything but there is so many things we are doing at the moment where the research base is less strong. Why is nobody questioning those things?” (Infectious Disease Physician, UK, 1704)
**Theme 2: Personal views about what evidence is current or applicable**
“I don't know what the evidence is supporting SDD- but... all the components of the bundle are all sort of evidence-based interventions, and so whoever on the Canadian Patient Safety Institute who put together the Safer Healthcare Now bundle for ventilator-associated pneumonia, obviously didn't consider SDD an important component, or must have felt that, you know, the evidence... I'm guessing- but for some reason they decided to not make that part of the bundle." (Infectious Disease Physician, Canada, 025)<br><br>“Should SDD come in as a recommendation from a national body, then it would become as high a priority as the sepsis bundle, as we currently understand it. So I think it would be a high priority; at the moment it’s not. (Critical Care Physician, UK, 4101)<br><br>"I guess one of the other issues that might show up as well, I'd say I remember about maybe ten years ago we would discuss it - I think the literature was still a bit more exciting at the time". (Critical Care Physician, Canada, 044)<br><br>“I mean, I think it’s... everyone’s aware of it but it’s kind of been a stagnant issue for, you know, ten years now”. (Critical Care Physician, Canada, 315)<br><br>“It is not high profile, there are other things that have come around now, maybe not high profile because units are using it because they totally signed up to it or is it like, we tried to dabble with it and got sort of knocked off our perch and we haven’t gone back to look at it again because other

things have come along." (Nurse, UK, 2681)<br><br>"You know, again I'm no expert on SDD, I must admit I haven't read everything – I've tried to keep abreast of it – but to me it almost seems to good to be true, and that's just my emotional response, right? And it just seems like a magic bullet.]"(Pharmacist, Canada, 104)<br><br>"We all see the same evidence whether it is in the literature or whether it is evidence with your own eyes, and yet your interpretation of it can be completely different based perhaps on whether or not you have got preconceptions." (Microbiologist, UK, 1704)<br><br>"So the paper we looked at was conducted in Europe, has different microbial flora, different patient population, and I guess the question was, you know, can you transfer these results to would we see this results in our unit." (Pharmacist, Canada, 013)<br><br>"[...] despite the evidence of this working, that people aren't using it, so there's something wrong with the evidence, there's something not convincing with the evidence, and it's probably for those reasons that I said before, that it doesn't have a similar patient population to what we have in Canada." (Critical Care Physician, Canada, 306)<br><br>"A cluster randomised controlled trial in appropriately chosen intensive care units in the UK. Because UK practice will differ from Dutch practice, from German practice and so on. So I would favour a UK cluster randomised study." (Infectious Disease Physician, UK, 1406)<br><br>"It would be very helpful and particularly if there is good research from the UK, because we use antibiotics differently, our ecology is different, our patients tend to be slightly different to other European ITUs and it also needs to include district hospitals like the one I am based on because we do definitely manage different complexity compared to specialist care centres." (Microbiologist, UK, 301)<br><br>"I think that is hard to answer because I think it depends on where you are geographically as much as anything else. I think SDD seems very popular in northern European countries, Scandinavian countries, maybe the Netherlands and I think there are areas that have got very low problems with VRE and MRSA rates and I think when you try and apply that maybe to north America and Southern Europe or even UK practice it becomes a little bit more difficult to try and identify the pros and cons, the risks versus the benefits of SDD." (Pharmacist, UK 3703)<br><br>"Well I think we'd need to see the evidence replicated you know in, in a setting outside of Northern Europe preferably in Australia and then depending on the nature of that evidence you know we would then approach it as we would our other clinical guidelines and you know think about implementing it as a, as a standard of practice." (Critical Care Physician, Australia, 301)
**Theme 3: Interpersonal and relational aspects of professional decision-making locally**
"I think there's also been a somewhat different approach by intensivists versus infectious disease physicians.[...] My sense is that there's also an ID versus critical care approach to the matter, and I tend to see differences between critical care colleagues and infectious disease colleagues that are often more tightly aligned with infection control." (Infectious Disease Physician, Canada, P03)<br><br>"I think that the reason, the main reason that we don't do it is because we are concerned that our microbiologists in the hospital are even more concerned about the possibility of resistant organisms even though there is some evidence that that doesn't happen but the microbiologists particularly are still concerned that it might happen and they don't want antibiotics sort of used in

any great amounts because of the possibility of resistance so I just had, just say to there that in our hospital our antibiotic usage is, is controlled by the microbiologists …" (Critical Care Physician, Australia, 304)

"Well I guess they would have more – well they just might have a different view of the evidence you know 'cause communities often do have different views of the same evidence and therefore – but also because you know their primary role is eradication of infections, they would see that as a greater priority than, than intensivists." (Critical Care Physician, Australia, 302)

" I mean the main people that worry about it are microbiologists. They do a ward round three times a week on ICU and sometimes you hear the comments "oh SDD again", you know, and one of our microbiologists is very much if he had way we wouldn't use it. I am not sure the others are so dogmatic but there is one definitely that would, I think, remove it if it was at all possible." (Pharmacist, UK, 604)

"... so everything is driven by... I shouldn't say "everything", most things are driven by the physicians, and by, you know, best practice, and what's current in the literature, and what they feel is, as a group of physicians, is best for the patients here in the Intensive Care Unit. So I'm not sure why that has not become part of our usual protocol of care. … This is the first time I've heard about it, so I don't know if this is something … that the physicians have discussed among themselves and decided that it's not right for us at this point, for whatever reason. I'm, you know, I'm not sure." (Nurse, Canada, 44)

"Only small [amount of influence over decision to implement SDD]. I mean I think the ultimate decision of taking this on will be with medical staff." (Nurse, UK, 4501)

"[...] adding something else like an SDD protocol that requires a certain commitment from the nurses to do this on a daily basis it would further tax the nursing staff. And so we've had initiatives in the past, too, that have failed because of nursing not buying into it." (Pharmacist, Canada, 114)

"So, they tend to push back every once in a while if they're not comfortable with something, so I think that that's a huge piece, to be able to engage those [nurses]." (Critical Care Physician, Canada, 307)

"So I think once, if that's agreed by, by the 6 organisations then I guess I wouldn't be surprised if it became a nurse kind of driven thing." (Nurse, UK, 4501)

"[...] at the highest levels are they willing to take this up and if it is then someone ends up writing a policy or procedure and that usually ends up being a nurse and then that gets endorsed by some sort of management committee." (Critical Care Physician, Australia, 202)

"For our own personal model here it means the team, which would be the intensivists and the pharmacists would say yes... or, review the evidence and say yes or no we're going to pursue this, we would bring it to our quality group - which is Nursing, Pharmacy, dietician, like, key multidisciplinary team - and then make a plan and action and then try to execute." (Pharmacist, Canada, 013)

"but the key to getting anything in place is to get some consensus and to get everyone's opinion and make sure that you tap all of the stakeholders so that they feel that they've had input, because ultimately your decision, if it's made, will only stick if people feel like they've had input into it."

(Pharmacist, Canada, 014)<br><br>"And especially since, you know, to... as a practice it's one of those things that it makes no sense for one doc to do it and another doc not to because, you know, the impact on the patient is over the course of their stay not, you know, I decide to start it on Friday and somebody stops it on Monday. So it's probably a departmental or a group decision to do that, if it's going to be implemented in any meaningful way, and I think people just aren't having that conversation given all those reasons." (Critical Care Physician, Canada, 044)<br><br>“I think we’d have to, we’d have to have consultation obviously with medical staff in the first instance to gain agreement. We struggle to implement anything that doesn’t share the unanimous support of the people who work in the unit. We would have to liaise with our infectious disease colleagues as well I think. I don’t think we could implement something like that without some involvement and support from them.” (Critical Care Physician, Australia, 305)<br><br>“I mean it’s a democracy so, anyone can institute changes but it’s done after discussion and agreement.” (UK 2001)<br><br>“And I think also we try where we can to do most things in a sort of, in a collegiate fashion with consensus rather than having one person implementing things.” (Critical Care Physician, UK, 304)
**Theme 4: An a priori commitment to future trials testing SDD**
“I think it is just wasting everybody’s time because I think the people that have got their mind set up against it, I am not sure there’s any other piece of …I mean I have worked with people that have said “one more study and I will maybe go for it” and then one more study would come out and they will always pick faults with the study, they will say “one more”, it is always one more and I just don’t think there is a study that could be designed that people would not pick fault with, that they would believe.” (Infectious Disease Physician, UK, 1704)<br><br>“We do a lot of research but I guess I can’t really see how that is research. It’s more implementation of a treatment which probably has evidence so you know maybe we should be doing that ourselves but I can’t really see how that’s, how that is a study.” (Critical Care Physician, Australia, 304)<br><br>"I would think so. The problem is that that sort of a study wouldn't actually help us in changing in our mind. I think... I mean, to randomize patients into that, I think we could do it, I don't think it would be that difficult. From a proof of concept to apply it to our unit and to get an idea of the actual amount of resources that would be needed, I think from those perspectives it would be a useful trial for us to enrol into. From the purpose of figuring out resistance patterns, that actually won't tell you anything, I don't think, because the resistance pattern of the whole unit changes as a unit, so randomizing people within the same unit I don't think would show any difference in resistance. I think at the end of that study it would say there was no difference in resistance between the two of them and I wouldn't believe it." (P14, Pharm)<br><br>“Mainly because I’ve looked at the evidence and I’m not convinced yet, myself, but I do think it is worth exploring on a large scale in a well-designed, multi-centre study. Yes, I would be willing to be part of that.” (Pharmacist, Canada, 113)<br><br>“Oh absolutely I think there’s, I think there’s equipoise on this issue and so [a trial] is absolutely justifiable.” (Infectious Disease Physician, Australia, 401)

“Yeah. I think it [a trial] is ethical because there is a huge, I think there is equipoise within the medical profession.” (Critical Care Physician, UK, 1701)
